# High-Intensity Interval Training Improves Cardiac Function by miR-206 Dependent HSP60 Induction in Diabetic Rats

**DOI:** 10.3389/fcvm.2022.927956

**Published:** 2022-06-29

**Authors:** Maryam Delfan, Raheleh Amadeh Juybari, Sattar Gorgani-Firuzjaee, Jens Høiriis Nielsen, Neda Delfan, Ismail Laher, Ayoub Saeidi, Urs Granacher, Hassane Zouhal

**Affiliations:** ^1^Department of Exercise Physiology, Faculty of Sport Sciences, Alzahra University, Tehran, Iran; ^2^Department of Exercise Physiology, Faculty of Sport Sciences, Alzahra University, Tehran, Iran; ^3^Department of Medical Laboratory Sciences, School of Allied Health Medicine, AJA University of Medical Sciences, Tehran, Iran; ^4^Department of Biomedical Sciences, University of Copenhagen, Copenhagen, Denmark; ^5^Department of Exercise Physiology, Faculty of Physical Education and Sport Sciences, University of Tehran, Tehran, Iran; ^6^Department of Anesthesiology, Pharmacology, and Therapeutics, Faculty of Medicine, University of British Columbia, Vancouver, BC, Canada; ^7^Department of Physical Education and Sport Sciences, Faculty of Humanities and Social Sciences, University of Kurdistan, Sanandaj, Iran; ^8^Division of Training and Movement Sciences, University of Potsdam, Potsdam, Germany; ^9^Movement, Sport, Health and Sciences Laboratory (M2S), UFR-STAPS, University of Rennes 2-ENS Cachan, Av. Charles Tillon, Rennes Cedex, France; ^10^Institut International des Sciences du Sport (2IS), Irodouer, France

**Keywords:** diabetes, apoptosis, miRNAs, exercise, cardiomyopathy

## Abstract

**Objective:**

A role for microRNAs is implicated in several biological and pathological processes. We investigated the effects of high-intensity interval training (HIIT) and moderate-intensity continuous training (MICT) on molecular markers of diabetic cardiomyopathy in rats.

**Methods:**

Eighteen male Wistar rats (260 ± 10 g; aged 8 weeks) with streptozotocin (STZ)-induced type 1 diabetes mellitus (55 mg/kg, IP) were randomly allocated to three groups: control, MICT, and HIIT. The two different training protocols were performed 5 days each week for 5 weeks. Cardiac performance (end-systolic and end-diastolic dimensions, ejection fraction), the expression of miR-206, HSP60, and markers of apoptosis (cleaved PARP and cytochrome C) were determined at the end of the exercise interventions.

**Results:**

Both exercise interventions (HIIT and MICT) decreased blood glucose levels and improved cardiac performance, with greater changes in the HIIT group (*p* < 0.001, η^2^: 0.909). While the expressions of miR-206 and apoptotic markers decreased in both training protocols (*p* < 0.001, η^2^: 0.967), HIIT caused greater reductions in apoptotic markers and produced a 20% greater reduction in miR-206 compared with the MICT protocol (*p* < 0.001). Furthermore, both training protocols enhanced the expression of HSP60 (*p* < 0.001, η^2^: 0.976), with a nearly 50% greater increase in the HIIT group compared with MICT.

**Conclusions:**

Our results indicate that both exercise protocols, HIIT and MICT, have the potential to reduce diabetic cardiomyopathy by modifying the expression of miR-206 and its downstream targets of apoptosis. It seems however that HIIT is even more effective than MICT to modulate these molecular markers.

## Introduction

Diabetes mellitus (DM) is a risk factor for cardiovascular disease that is estimated to affect more than 439 million people by 2030 (1). Of note, patients are at increased risk of developing diabetic cardiomyopathy (DCM) which is unrelated to risk factors such as hypertension, coronary artery disease, and valvular heart disease (2). DCM has been associated with disturbances in the myocardial structure and function, involving left ventricular hypertrophy and dysfunction, myocardial fibrosis, and cellular signaling pathway abnormalities, eventually leading to heart failure (3).

The available research suggests that the inhibition of molecular signaling pathways involved in DCM may mitigate the pathological changes in cardiomyocytes of patients with diabetes (2). For instance, high glucose appears to induce early cellular changes, including apoptosis, in cardiomyocytes in DCM (4). Apoptosis and cell survival signals in diabetic cardiomyocytes may be partly mediated by microRNAs (miRNAs) that regulates gene expression post-transcriptionally (5). Cardiac/skeletal muscle-specific miR-206 has been suggested to be involved in apoptotic processes, and over-expression of miR-206 may even accelerate cardiomyocyte apoptosis by ultimately leading to DCM (6, 7). Hyperglycemic conditions up-regulate miR-206 in diabetic heart disease (6). In turn, miR-206 negatively regulates heat shock protein 60 expression (HSP60), which serves as a protective component against diabetes-induced cardiac damage and apoptosis (3).

Physical activity and exercise are effective means to treat diabetes and its cardiovascular adverse effects (8). Recommendations of the World Health Organization (WHO) suggest a minimum weekly dosage of 150 min of moderate to vigorous intensity physical activity (30 min, 5 days per week) to prevent and improve diabetic adverse effects (9). There is evidence however, that large proportions of patients do not meet WHO guidelines (10) and ~50% of diabetics do not participate in any physical activity, according to a research (11). Accordingly, new intervention strategies are needed that are attractive for individuals and effective in terms of health outcomes at the same time. Patients, especially those with T1DM do not take up exercise due to fear of hypoglycemia and also lack of time (12). Recent evidence indicates that high-intensity interval training (HIIT) has the potential to reduce blood glucose levels (hypoglycemia) which is an established effect for moderate-intensity continuous training (MICT) (13, 14). Moreover, HIIT produces similar or greater improvements in cardiovascular function, hyperglycemia, and other adverse events of diabetes even with 60–80% less exercise time compared with MICT (13, 15). Thus, HIIT may be considered a viable and time-efficient alternative to MICT.

The molecular signaling processes associated with DCM have recently received much attention. While the mechanisms by which hyperglycemia effects cardiomyocytes are not fully elucidated, it has been demonstrated that excessive glucose triggers cellular death by increasing miR-206 expression (6). Accordingly, we examined the effects of different glucose concentrations on C2C12 skeletal muscle cells to examine the differential effects of HIIT and MICT on the expression of miR-206 in DCM by monitoring apoptosis-related markers, HSP60, and cell viability. To better understand the mechanisms underlying apoptosis and exercise-induced cardioprotection against apoptosis, we tested the following hypotheses based on the available literature (6, 16): (1) High glucose concentrations *in vitro* up-regulate the expression of miR-206, leading to decreased HSP60 and increased apoptosis-related markers, (2) Both HIIT and MICT down-regulate the expression of miR-206 and apoptotic markers and up-regulate HSP60 levels, and (3) HIIT produces greater benefits in the molecular and functional markers of DCM.

## Materials and Methods

### Animals

Male Wistar rats (8 weeks old, *n* = 18), weight 260 ± 10 g, were acquired from the Pasteur Institute (Tehran, Iran). The rats were kept in a room with 50% humidity, a temperature of 22°C, a 12 h light/12 h dark cycle, and had access to laboratory chow and water *ad libitum*. All animal procedures were carried out humanely and in accordance with the guidelines of the Tehran University of Medical Sciences' Animal Care Committee (EC-00312).

### Induction of Diabetes

Rats received STZ (55 mg/kg IP; Sigma-Aldrich, St. Louis, MO) in a 2% solution of cold 0.1 M citrate buffer (pH 4.5) to induce type 1 diabetes (17, 18). Blood glucose levels were measured using a glucometer (Glucocard 01, Japan) 3 days after STZ treatment to confirm diabetes. Rats were considered to be diabetic when blood glucose concentrations were higher than 300 mg/dL (6, 18).

### Training Protocol

Diabetic animals were randomly allocated to three groups (*n* = 6 in each group): control, MICT (Moderate-Intensity Continuous Training), and HIIT (High-Intensity Interval Training). The training was conducted 5 days per week for 5 weeks. After having familiarized the animals to the treadmill, the velocity at VO_2max_ (vVO_2max_) was assessed every week by using a modified ramp test protocol as previously reported (19, 20).

#### HIIT

The HIIT program consisted of 5 min of a warm-up with running at 30–40% of vVO_2max_, followed by the main training session of 3 min of running at 85–90% of vVO_2max_ (the running speed was increased from 12 m/min during the first week to 24 m/min during week five), followed by 1 min of recovery. This cycle was performed four times per session and ended with a 5 min cool-down at 30–40% of vVO_2max_ (Table 1).

**Table 1:** Intensity of HIIT (in meters per minute) based on vVO_2max_.

**Exercise progression**	**Main aspects of training**			
**Training components**	**Warm up**	**High intensity**	**Recovery**	**Cool down**
Time of training	5 min	3 min	1 min	5 min
Intensity of training (% of vVO_2max_)	30–40%	85–90%	30–40%	30–40%
First week (m/min)	4.5–6	12.5–13.5	4.5–6	4.5–6
Second week (m/min)	5.5–7.5	16–17	5.5–7.5	5.5–7.5
Third week (m/min)	6.5–8.5	18.5–20	6.5–8.5	6.5–8.5
Fourth week (m/min)	7–9.5	20.5–21.5	7–9.5	7–9.5
Fifth week (m/min)	8–11	23–24	8–11	8–11

*The slope of the treadmill was set at 0° at all stages of training. HIIT, High-Intensity interval training; vVO_2max_, the velocity at VO_2max_*.

#### MICT

The MICT protocol consisted of 40 min of continuous running. Every session included 5 min of a warm-up with running at 30–40% of vVO_2max_, followed by 30 min of running at 60–65% of vVO_2max_ (the running speed was increased from 9 m/min during the first week to 18 m/min in week 5), and ended with 5 min cool down at 30–40% of vVO_2max_ (Table 2). Exercise volume was similar between the intervention groups.

**Table 2:** Intensity of MICT (in meters per minute) based on vVO_2max_.

**Exercise progression**	**Warm up**	**Main part of training**	**Cool down**
Time of training	5 min	30 min	5 min
Intensity of training (% of vVO_2max_)	30–40%	60–65%	30–40%
First week (m/min)	4.5–6	9–9.5	4.5–6
Second week (m/min)	5.5–7.5	11.5–12	5.5–7.5
Third week (m/min)	6–8.5	12.5–13.5	6.85
Fourth week (m/min)	7.5–10	15–16	7.5–10
Fifth week (m/min)	8–10.5	16–17.5	8–10.5

*The slope of the treadmill was set at 0° at all stages of training. MICT, Moderate-Intensity continuous training; vVO_2max_, the velocity at VO_2max_*.

#### Control

Animals in the control group did not participate in any exercise intervention, but were positioned on a stationary treadmill for 10–15 min daily to simulate the environmental conditions of the exercise protocols.

### Echocardiography and Left Ventricular Extraction

Animals were anesthetized with intraperitoneal injections of ketamine (90 mg/kg body mass) and xylazine (10 mg/kg body mass) before investigating cardiac function *in vivo*. After shaving the anterior chest, the animals were positioned in the left lateral decubitus position and a rectal temperature probe was inserted so that the body temperature could be maintained between 37 and 37.5°C using a heating pad. Conventional two-dimensional M-mode echocardiography images (GE Vingmed Ultrasound, Horton, Norway) were obtained to assess markers of cardiac function. Left ventricular end-diastolic dimensions and ejection fractions were assessed using a 10-MHz probe (Horton, Norway) in the short axis (papillary) views. At least three cardiac cycles were used to conduct echocardiographic measurements. All echocardiograms were completed by one operator who was blind to genotype and treatment, using specialized software (EchoPac V113.05, GE Healthcare, Horton, Norway). Blood samples were taken directly from rat hearts, and serum was obtained after centrifugation at 3,000 g for 4 min at 10°C. In addition, left ventricular tissue was removed and washed in saline before promptly frozen in liquid nitrogen.

### Cell Culture

A mouse skeletal myoblast cell line (C2C12), which was purchased from Iran's Pasteur Institute, was maintained in Dulbecco's modified Eagle medium (DMEM) supplemented with 20% heat-inactivated fetal bovine serum (FBS) in growth medium (GM) and 1% penicillin-streptomycin. DMEM containing 2% heat-inactivated horse serum (differentiation medium, DM) and 1% penicillin-streptomycin was utilized for myogenic differentiation from myoblasts into myotubes. The MTT assay was used to evaluate cell viability as previously described (21).

### microRNA Mimic, and Antisense microRNA Transfection

The miR-206 sequences were transfected into C2C12 cells using RNAiMAX (Invitrogen) according to the manufacturer's instructions. Antisense (2′-*O*-methyl) oligonucleotides in a serum-containing medium were transfected into C2C12 cells. Serum depletion was used to differentiate the cells into muscle cells after 72 h.

### miRNA Expression by Real-Time-PCR

MicroRNA was extracted using a miRNeasy mini kit (Qiagen, Hilden, Germany) using the manufacturer's instructions. MiScript II RT kit (Qiagen, Hilden, Germany) was used to synthesize cDNA of the miR-206 genes. Polymerase chain reaction (PCR) was performed using a Real-Time PCR machine (Corbett Rotor-Gene 6000, Qiagen, Hilden, Germany). The Real-Time program for miR-206 (miR-206 miScript Primer Assay, Qiagen, Hilden, Germany) was based on the miScript SYBR Green PCR Kit (Qiagen, Hilden, Germany), which included a cycle of 95°C for 15 min, followed by 40 cycles of 94°C for 15 s, 55°C for 30 s, and 70°C for 30 s. SNORD-61 (SNORD 61 miScript Primer Assay Qiagen, Hilden, Germany) was used to normalize target gene transcript levels (22). The 2 ^−ΔΔCT^ (delta-delta Ct) method was used to convert real-time CTs to quantitative data.

### Western Blot Analysis

Cellular protein was isolated from previously frozen left ventricular tissue by homogenizing 70–100 mg of tissue in a modified RIPA buffer (50 mm Tris–HCl, pH 7.4, 1% Triton X-100, 0.2% sodium deoxycholate, 0.2% SDS, 1 mm Na-EDTA, and 1 mm PMSF) supplemented with protease inhibitor cocktail and PMSF (Roche, Mannheim, Germany). Protein concentration was determined with a Bradford assay, and equivalent amounts of protein were subjected to SDS–PAGE and then transferred onto a PVDF membrane. The membranes were then incubated for 2 h at room temperature in blocking buffer (1: Tris-buffered saline (TBS), 0.5% Tween-20, and 5% non-fat dry milk). Blots were incubated overnight at 4°C with primary antibodies against cleaved PARP, cytochrome C, HSP60 (Cell Signaling Technology, Beverly, MA, USA), and β-actin (Abcam, Cambridge, MA, USA). We utilized an enhanced chemiluminescent substrate (ECL) after incubating with second HRP-conjugated antibodies to visualize protein bands. Densitometry was used to analyze band densities using Image J software.

### Statistical Analyses

Results are presented as mean ± SEM (standard error of the mean). The Kolmogorov-Smirnov (KS) test was used to assess data normality. The Levene test was applied to establish variance homogeneity. After confirmation of data normality and homogeneity, a one-way analysis of variance (ANOVA) was conducted to verify significant differences between groups. If ANOVA revealed significant outcomes, the Tukey *post-hoc* test (least significant difference) was computed for pair-wise comparisons to identify the potential difference between three groups. Partial eta-squared (η^2^) was used as an effect size measure. The statistical significance level for all comparisons was set at *p* < 0.05. Statistical analysis was carried out using SPSS 19 (IBM SPSS, United Kingdom).

## Results

### General Characteristics of Animals

**Figure 1:**
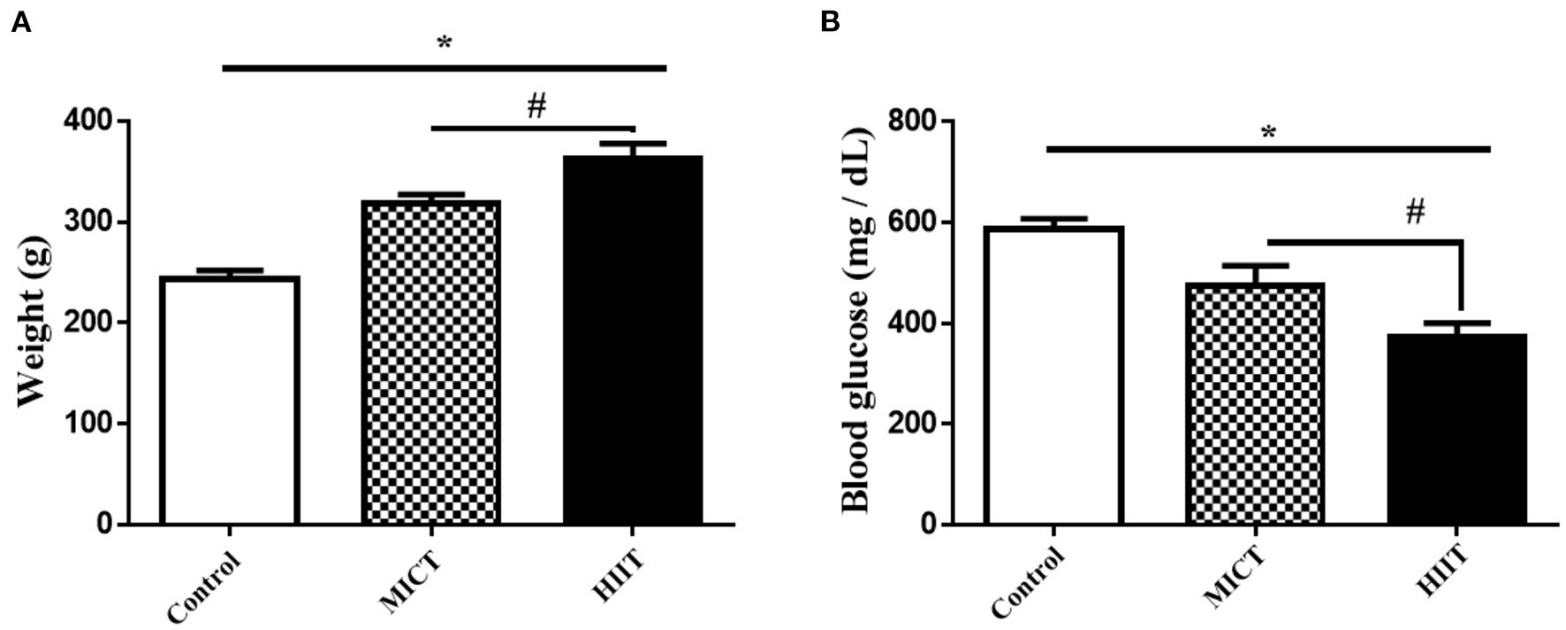
Body mass and fasting blood glucose concentrations in the three experimental groups. **(A)** Body mass **(B)** Blood glucose. Data are presented as means ± standard error of the mean (SEMs). *Significant differences between control and intervention groups, ^#^Significant differences between MICT and HIIT. MICT, moderate-intensity continuous training; HIIT, high-intensity interval training. Rats (*n* = 6 per group) were trained for 5 weeks.

The general characteristics of the animals used in this study are shown in Figure 1. There were significant differences in body mass between groups (*F*_2,15_ = 167.053, *p* < 0.001, η^2^: 0.957), with differences in body mass between the MICT and HIIT groups (*p* < 0.003). There were also significant between group differences in glucose levels (*F*_2,15_ = 74.625, *p* < 0.001, η^2^: 0.909). Blood glucose levels were significantly lower in the HIIT and MICT groups compared with the control group (36.5 and 18.9%, respectively, *p* < 0.001). Glucose levels were significantly lower in the HIIT group compared with the MICT group (*p* < 0.001) as shown in Figure 1B.

### *In vivo* Left Ventricular Function

The mean left ventricular end-diastolic diameters in control rats were significantly greater than that observed in the MICT and HIIT groups as shown by M-mode echocardiograms (7.56 ± 0.41 vs. 6.20 ± 0.40 and 5.78 ± 0.41 mm, respectively, *p* = 0.01; Table 3). Moreover, the mean left ventricular end-systolic diameters in both intervention groups (MICT and HIIT) were significantly lower compared with the control group (3.1 ± 0.17 and 2.90 ± 0.14 vs. 4.49 ± 1.12 mm, *p* = 0.002). In addition, there were differences in ejection fractions between control and exercise animals (66.67 ± 0.93 vs. 75.59 ± 3.71% and 80.38 ± 2.63%; *p* = 0.003). These findings indicate that both exercise protocols (MICT and HIIT) improved heart function in diabetic animals. Further, there were differences in all electrocardiography parameters (LVESD, LVEDD, and ejection fraction; *p* = 0.002) between the MICT and HIIT groups. Overall, our results indicate that the HIIT protocol enhanced cardiac function more than the MICT protocol.

**Table 3:** Echocardiographic changes produced by exercise in diabetic rats.

**Variable**	**Control**	**MICT**	**HIIT**
LVEDD (mm)	7.56 ± 0.41	6.20 ± 0.42^*^	5.78 ± 0.41^*^#
LVESD (mm)	4.49 ± 1.12	3.10 ± 0.17^*^	2.90 ± 0.14^*^#
Ejection fraction (%)	66.67 ± 0.93	75.59 ± 3.71^*^	80.38 ± 2.63^*^#

Data presented as means ± SEMs ^*^Significant differences between control and exercise groups, #Significant differences between MICT and HIIT. MICT, Moderate-Intensity continuous training; HIIT, High intensity interval training; LVEDD, Left ventricular end-diastolic diameter; LVESD, Left ventricular end-systolic diameter.

### HIIT Decreases miR-206 Expression

**Figure 2:**
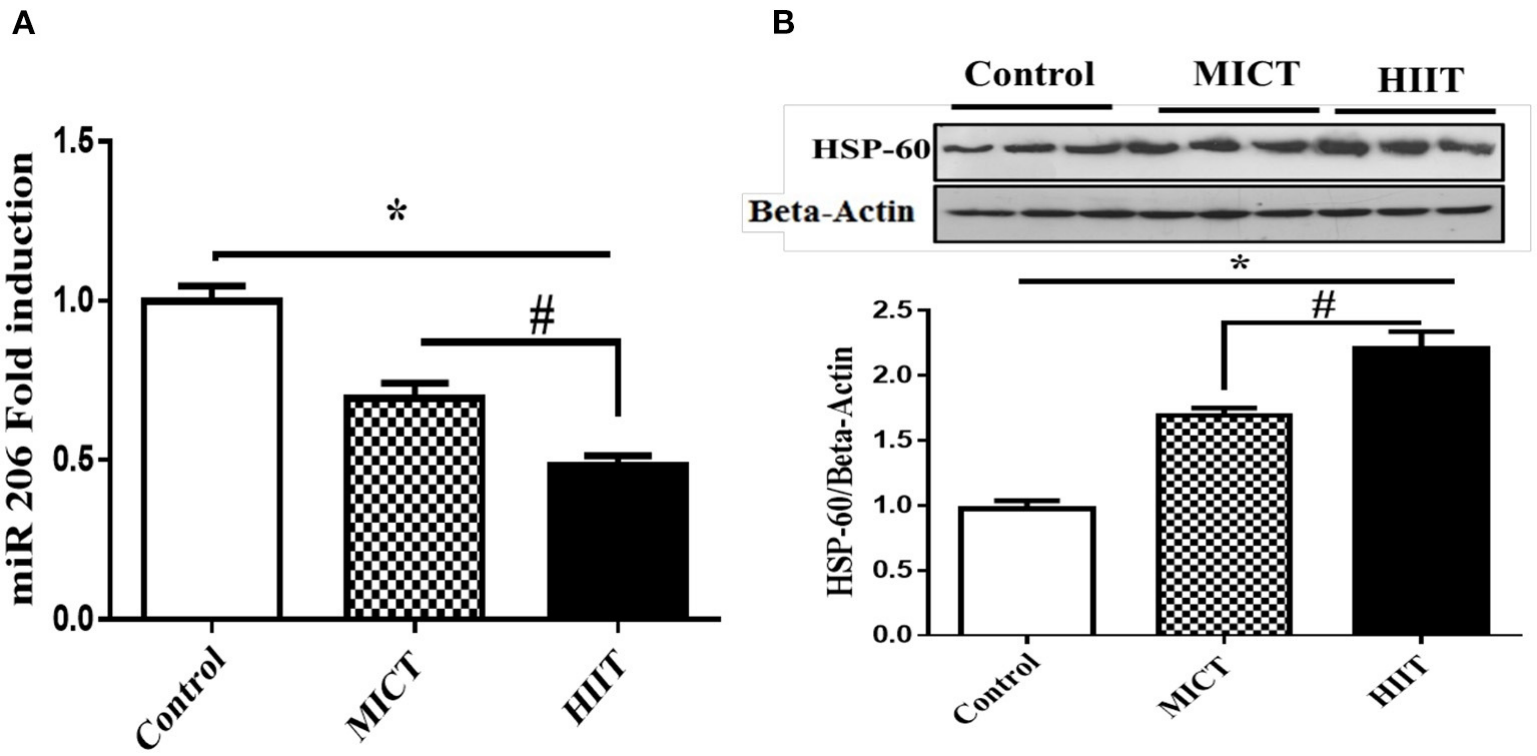
Effects of exercise on miR-206 mRNA. **(A)** HSP60 protein expression. **(B)** Data are presented as means ± standard error of the mean (SEMs). *Significant differences between control and intervention groups, ^#^Significant differences between MICT and HIIT. MICT, moderate-intensity continuous training; HIIT, high-intensity interval training; *n* = 6 and rats trained for 5 weeks.

We measured the expression of miRNA in the left ventricular tissue to better understand the potential molecular mechanisms for the beneficial effects of physical exercise on cardiac parameters. Both exercise protocols significantly reduced miR-206 expression compared with control (*F*_2,15_ = 222.149, *p* < 0.001, η^2^: 0.967). MICT and HIIT protocols reduced miR-206 expression by 30.3 and 51.3%, respectively. In addition, HIIT showed a 1.7 fold larged reduction in miR-206 expression compared with MICT (*p* < 0.001; Figure 2A).

### HIIT Enhances HSP60 Expression at the Protein Level

The expression levels of HSP60 protein were determined to investigate potential molecular links between physical exercise and apoptosis in cardiac cells. Both MICT and HIIT protocols enhanced HSP60 expression (1.68- and 2.21-fold, respectively) (*F*_2,15_ = 306.781, *p* < 0.001, η^2^: 0.976). HIIT resulted in a ~50% greater increase in HSP60 expression compared with MICT (*p* < 0.001; Figure 2B).

### HIIT Program Mitigates Diabetes-Induced Ventricular Apoptosis

**Figure 3:**
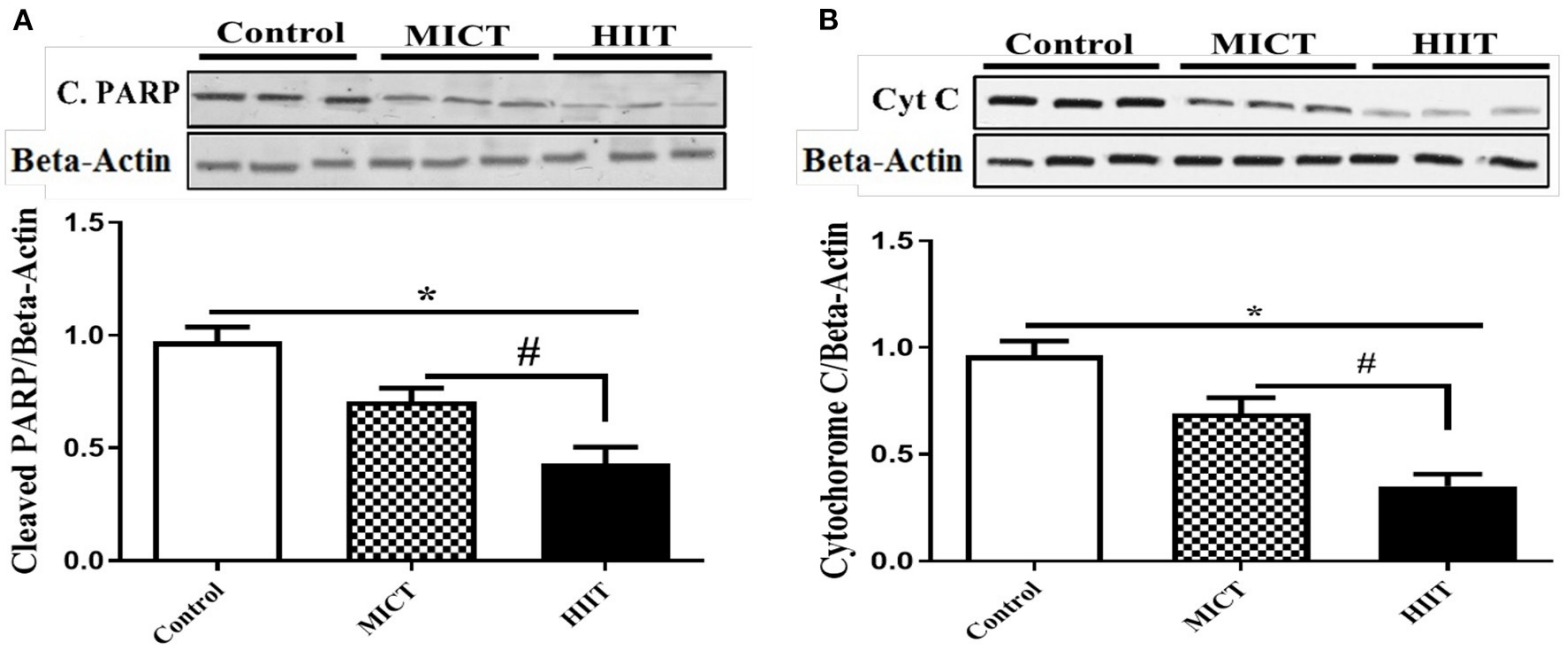
Effects of HIIT and MICT protocol on apoptotic intermediate molecules. **(A)** Cleaved PARP (C.PARP). **(B)** Cytochrome C. Data are presented as means ± standard error of the mean (SEMs). *Significant differences between control and intervention groups, ^#^Significant differences between MICT and HIIT. MICT, moderate-intensity continuous training; HIIT, high-intensity interval training, *n* = 6 and rats trained for 5 weeks.

We analyzed the protein expression of cleaved PARP and cytochrome C to investigate the effects of the HIIT and MICT on key intermediates of the apoptosis pathway. The expressions of apoptotic marker (PARP and Cyt C) were reduced in both exercise groups (p <0.001), with HIIT reducing cleaved PARP (*F*_2,15_ = 101.337, *p* < 0.001, η^2^: 0.931) (Figure 3A), and cytochrome C protein levels (*F*_2,15_ = 129.552, *p* < 0.001, η^2^: 0.945) (Figure 3B) more than MICT. Our results indicate that both exercise protocols reduced protein markers of apoptosis in diabetic rat hearts, with greater improvements produced by HIIT compared to MICT.

### High Glucose Induces miR-206 Expression and Reduces Cell Viability

**Figure 4:**
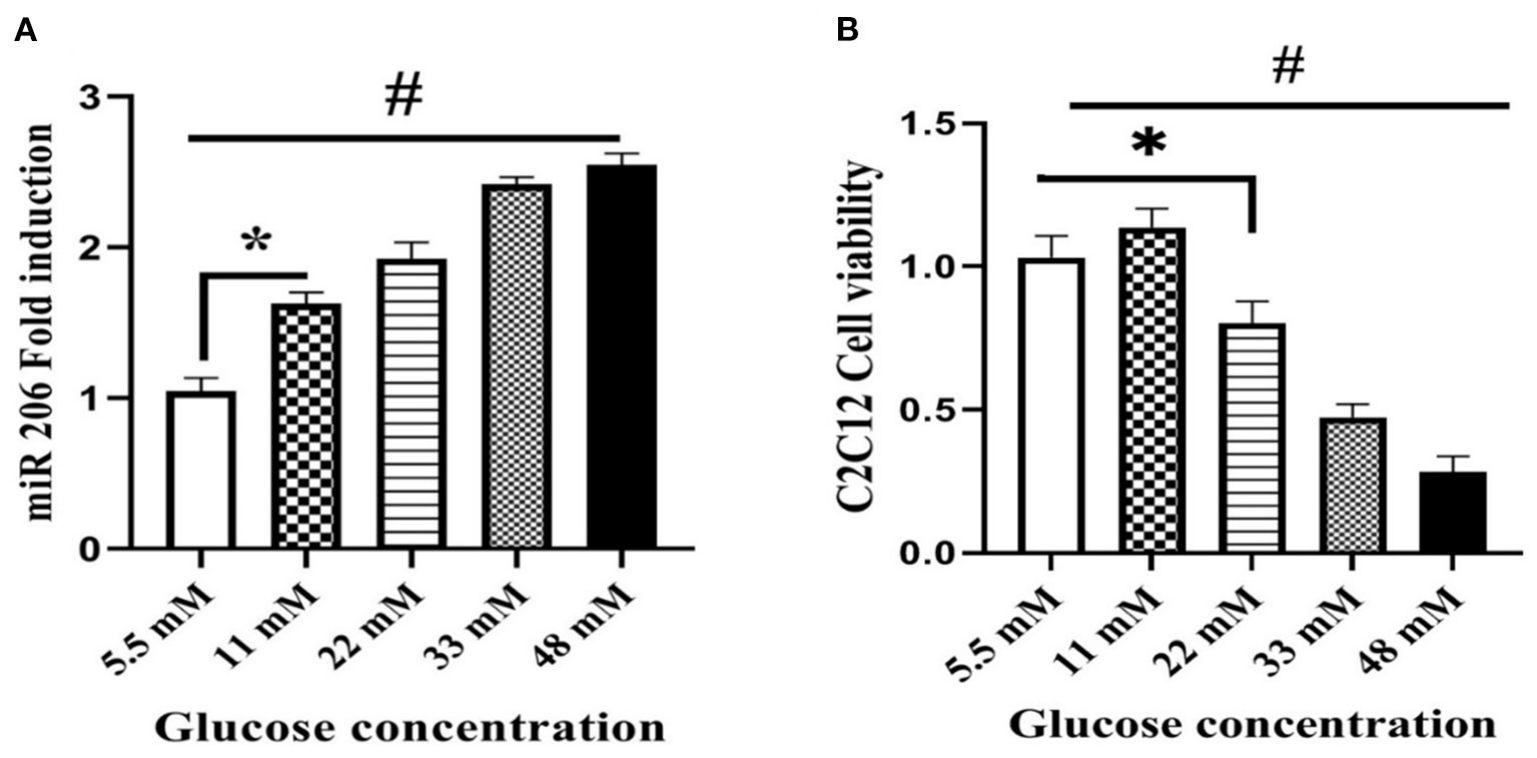
Effect of different glucose concentrations on miR-206 expression and viability of C2C12 cells. **(A)** miR-206 expression **(B)** cell viability. Data are presented as means ± standard error of the mean (SEMs). *n* = 6. C2C12 cells treated for 24 h with high glucose DMEM media, *Significant differences between normal glucose and high glucose, and ^#^significant differences between different glucose concentrations.

**Figure 5:**
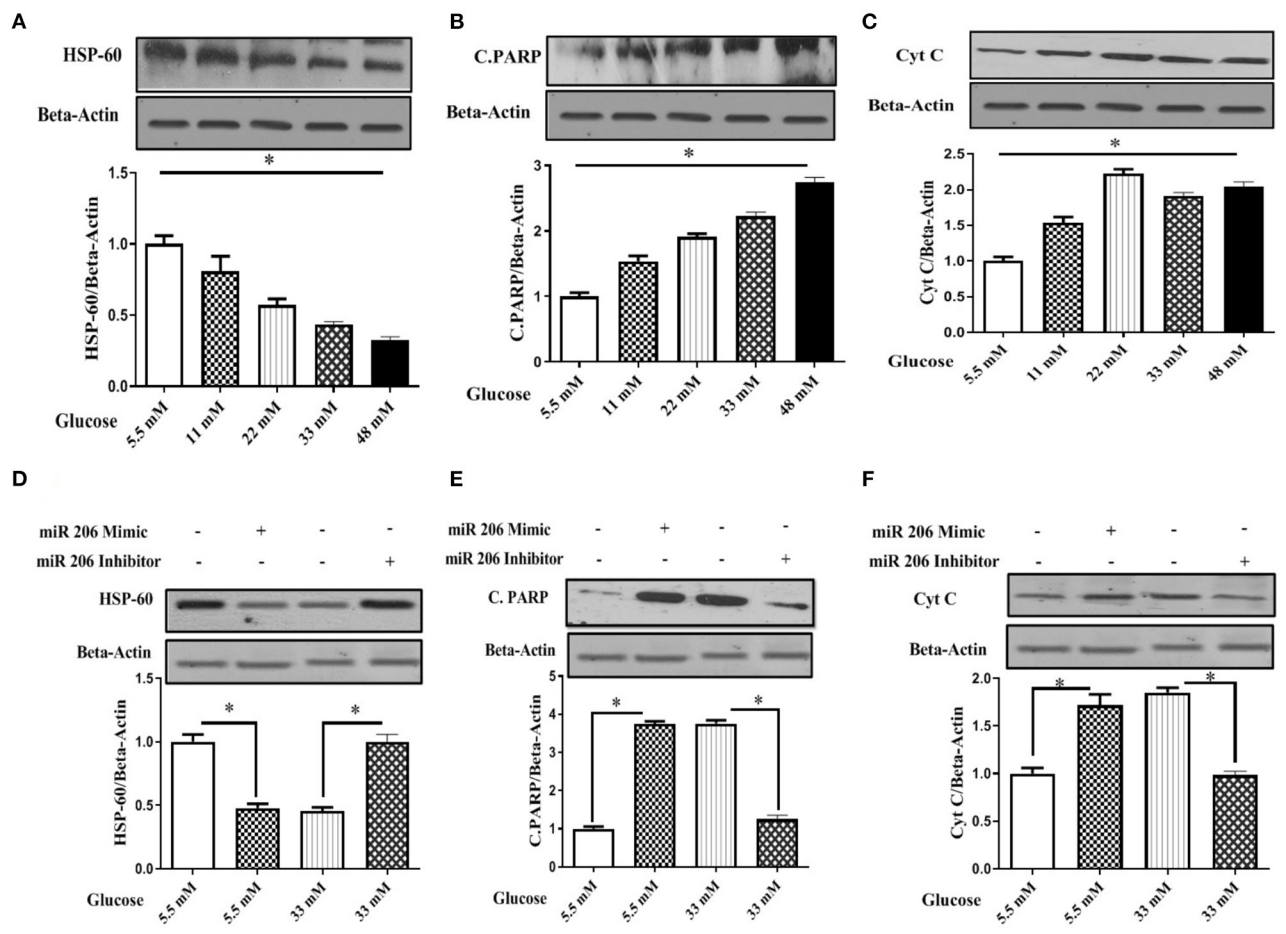
Effect of different glucose concentrations and miR-206 modulation on HSP60 and apoptosis markers in C2C12 cells **(A)** HSP60 protein expression, **(B)** Cleaved PARP (C.PARP) protein expression, **(C)** Cytochrome C protein expression, **(D)** Effects of miR-206 mimic and inhibitor on HSP60 protein expression, **(E)** Effects on miR-206 mimic and inhibitor on C-PARP protein expression, **(F)** Effects of miR-206 mimic and inhibitor on Cytochrome C protein expression. *n* = 5, C2C12 cells treated for 24 h with high glucose DMEM media. *Significant differences.

We treated C2C12 cell lines with increasing concentrations of glucose (5.5, 11, 22, 33, and 48 mM) to determine a potential role of hyperglycemia on miR-206 expression. Increasing concentrations of glucose enhanced miR-206 expression in a dose-response manner (*F*_4,25_ = 339.240, *p* < 0.001; Figure 4A). In addition, the viability of C2C12 cells was evaluated in the presence of different glucose concentrations by using the MTT assay. Increasing concentrations of glucose reduced cell viability in a dose-dependent manner at glucose concentrations greater than 11 mM (*F*_4,25_ = 182.212, *p* < 0.001; Figure 4B). We also assessed the effects of different concentrations of glucose on HSP60 and protein markers of apoptosis. Increases in glucose produced concentration-dependent reductions in HSP-60 protein (*F*_4,20_ = 107.874, *p* < 0.001), and increases in cleaved PARP (*F*_4,20_ = 533.231, *p* < 0.001) and cytochrome C (*F*_4,20_ = 293.547, *p* < 0.001) protein expression levels (Figures 5A–C).

We modulated miR-206 expression and analyzed apoptosis markers to provide additional evidence for the role of miR-206 role in high-glucose-induced myocyte apoptosis. C2C12 cells were transfected with a miR-206 mimic sequence and harvested in the presence of 5.5 and 33 mM of glucose. The miR-206 mimic reduced HSP60 protein expression and induced the protein expression of apoptotic markers in the presence of normal glucose. However, the transfection with the miR-206 antisense sequence (inhibitor) reversed high glucose (33 mM) reduced the expression levels of HSP-60 (*F*_3,16_ = 215.402, *p* < 0.001), and increased cleaved PARP (*F*_3,16_ = 1,866.64, *p* < 0.001), and the cytochrome C (*F*_3,16_ = 200.5.6, *p* < 0.001) expression levels (Figures 5D–F).

## Discussion

Physical exercise has long been considered an effective cardioprotective strategy that improves the anatomical and functional abnormalities of the diabetic heart (8). Our study compared the effects of two different exercise protocols (high intensity interval training [HIIT] vs. moderate-intensity continuous training [MICT]) on cardiac function and apoptosis signaling pathways, focusing on miR-206, HSP60, and apoptosis-related markers. The main findings of this study were that: (1) both HIIT and MICT improved blood glucose levels and cardiac function, with the HIIT protocol producing greater benefits, and (2) both exercise protocols reduced the expression of miR-206 expression and apoptotic markers and increased the expression of HSP60, while HIIT induced greater changes than MICT.

Cardiomyocyte apoptosis is an underlying component of various cardiac diseases, including in high glucose-induced cardiotoxicity (3). MicroRNAs, or small non-coding RNAs regulating gene expression, are potential diagnostic and therapeutic molecules in diabetic individuals with cardiovascular disease. High glucose concentrations increase the expression of miRNAs, such as miR-1 or miR-206, in C2C12 cells and rat cardiac myocytes (4, 23). Our *in vitro* findings suggest that miR-206 may be a destructive miRNA that activates apoptosis signaling pathways. In support of this are the findings of Shan et al. who reported that treatment of cardiomyocytes with 25 mM glucose induced apoptosis via miR-1/miR-206, and moreover, that these microRNAs bind to HSP60, an anti-apoptotic protein, thereby downregulating HSP60 expression (6). Our findings also suggest that exposure to high glucose concentrations induced miR-206 expression, increased cytochrome C and cleaved PARP protein expression, and inhibited HSP60 protein expression in C2C12 cells. On the other hand, induction of miR-206 with a mimic sequence inhibited the expression of HSP60 at normal glucose concentrations, and the miR-206 antisense sequence reversed the inhibitory effects of a high glucose concentration (33 mM) on HSP60 and promoted HSP60 protein expression.

HSP60 is present in different intracellular compartments, including the cytosol, plasma-cell membrane, extracellular space, mitochondria, and also in the circulation (24). The localization of HSP60 is an important consideration, as extracellular HSP60 can activate the innate immune system *via* Toll-like receptor (TLR)-4 and increase cardiac myocyte apoptosis, while HSP60 acts as an anti-apoptotic factor when present in the cytosol or mitochondria (25). Cytoplasmic HSP60 binds to Bax and Bak (pro-apoptotic proteins) and inhibits their integration into the mitochondrial membrane, thereby indirectly limiting the activation of mitochondrial cytochrome C and the apoptotic cascade (26). Additionally, our findings demonstrate that a miR-206 mimic enhanced protein expression of apoptotic markers at normal glucose concentrations, whereas miR-206 inhibitors decreased the expression of apoptotic markers at higher glucose concentrations. Therefore, miR-206 acts, at least to some extent, targets the expression of cytochrome C and cleaved PARP, two apoptosis-related proteins, and inhibits HSP60 (an anti-apoptotic protein). Our results reinforce the concept that increased miR-206 expression can contribute to high glucose-induced cell death.

The balance between cell survival and cell death signals is complex, particularly in muscle cells. We hypothesized that high glucose concentrations detrimentally impact cell survival; thus, we investigated the adverse effects of high glucose concentrations on cell viability. Concentrations of glucose >11 mM decreased cell viability, which can be attributed to increases in miR-206 and apoptotic-related biomarkers, as well decreases in HSP60 expression. However, cell viability was not affected at 11 mM glucose despite increases in miR-206 and apoptotic markers. Cell viability in 11 mM glucose may be interpreted as a protective mechanism involved in cell survival at lower glucose concentrations, but this remains untested as only a few studies examined the cellular consequences of various concentrations of glucose. Our findings are supported by previous *in vitro* studies indicating that intermittent high glucose concentrations exacerbate oxidative stress and apoptosis in endothelial cells (27).

Our study indicates that HIIT-induced improvements in blood glucose levels and cardiac function were superior than the effects of MICT in STZ-induced diabetic rats. Higher intensity exercise protocols induce greater improvements in glycemic control when compared to moderate-intensity protocols (28, 29). A study by Chavanelle et al. reported that HIIT induced greater insulin-stimulated Akt phosphorylation and muscle glucose transporter 4 (GLUT4) expression than MICT, implying that HIIT is more efficient in improving glucose metabolism (28). The impact of the two different exercise protocols on glycemic control is probably attributed to differences in the effects of the skeletal muscle activity on stimulated glucose transport (30). Moreover, enhanced hepatic glucose production is a key contributor to hyperglycemia, and HIIT reduces hepatic glucose production following exercise more than MICT (31). In addition, high-intensity exercise depletes muscle glycogen concentrations to a greater extent than low-intensity exercise (32). It is probable that exercise-induced glycogen depletion stimulates larger increases in glucose uptake to replenish glycogen stores. Our study confirms these expectations in STZ-induced diabetic rats, where HIIT was superior to MICT in improving blood glucose metabolism (Figure 1B). Our results are also consistent with the findings of Terada et al. who reported that HIIT caused greater reductions in blood glucose levels than MICT in patients with type 2 diabetes (T2DM) after 24 h of exercise (33). Similarly, Mendes et al. observed that HIIT had a greater impact on glycemic control than MICT in patients with T2DM treated with metformin (34).

Other noteworthy findings from our study included the down-regulation of miR-206 in the left ventricular tissues of both exercise groups, HIIT reduced miR-206 expression by ~1.7-fold more times than the effects of MICT (Figure 2). Exercise is a powerful regulator of miRNAs expression, whose expression depends on specific training parameters such as intensity, duration, and volume (35). Since high glucose concentrations increased miR-206 expression *in vitro*, it can be inferred that lowering glucose levels, which is a goal of prescribing exercise for patients with diabetes, could also decrease miR-206 following exercise, specifically after high-intensity training. Preliminary studies demonstrated an association between pathological heart conditions and changes in myosin heavy chain (MyHC) proteins, with transformation of the faster α-MyHC isoform to the slower β-MyHC isoform (36). Elevated miR-206 expression correlates with pathologic increases in β-MyHC expression in the left ventricle, and miR-206 deletion leads to conversion of slow to fast muscle MyHC isoforms (37). While the mechanisms by which exercise protects cardiomyocytes from various cardiac insults are unknown, it has been proposed that exercise increases α-MyHC protein expression and suppresses the isoform shift to β-MyHC in the left ventricle, hence aiding in the improvement of cardiac function (38). It should be noted that a recent study reported no differences in the expression of miR-206 before and after physical exercise (39). This discrepancy in findings could be attributed to the induction of diabetes in the current study, the exercise modes, intensity, and duration of the intervention.

Cardiac and skeletal muscle performance is highly dependent on ATP-generating pathways for energy supply during exercise, and signaling during high-intensity training may be the consequence of alterations in the intracellular environment during training adaptations. For example, the modulated expression of miR-206 can stimulate AMP-activated protein kinase (AMPK), a cellular metabolite-sensing protein kinase (40). The AMPK signaling pathway is activated during physical activity in an intensity-dependent manner in response to fluctuations in the cellular energy states, thereby triggering carbohydrate metabolism to replenish ATP levels (41). Another key finding of our study was that both exercise training protocols enhanced HSP60 expression in the left ventricular tissues, with the HIIT protocol causing a ~50% greater expression of HSP60 MICT (Figure 2B). Overexpression of the chaperone protein HSP60 can boost electron transport chain activity and ATP generation in injured cardiomyocytes (42). HIIT improved the expression of HSP60 more than MICT likely because exercise enhances ATP turnover in an intensity-dependent manner. The main mechanisms driving the effect of exercise on apoptosis may include metabolic perturbations (e.g., oxidative stress, low pH, muscle temperature, ROS, glycogen depletion, and lactate concentration) experienced during the exercise, particularly with HIIT (43). It is likely that these metabolic challenges following training increase the expression of HSP60 as HSPs respond to stress. Another postulated mechanism is that miR-206 contributes to cardiomyocyte apoptosis by post-transcriptional repression of insulin-like growth factor 1 (IGF-1) (16). Additionally, reduced HSP60 suppresses the IGF-1 signaling pathway, resulting in diabetic cardiomyopathy (44). On the other hand, exercise acts enhances the IGFI/PI3K/Akt survival pathway in diabetic hearts (45).

Our findings indicate that the expression of apoptotic markers was reduced in the left ventricular tissues of both training groups, with HIIT decreasing cleaved PARP and cytochrome C protein levels more than MICT. A growing body of evidence indicates that mitochondrial dysfunction may be a key determinant of oxidative stress and consequently, myocardial apoptotic signaling in pathological conditions (3). In particular, exercise protects against the activation of apoptotic cascades by improving mitochondrial function and structure. For example, Veeranki et al. reported that exercise ameliorates mitochondrial transmembrane potential and prevents cytochrome C leakage into the cytoplasm, reducing cardiomyocyte apoptosis in *db/db* mouse hearts (46). The intensity of exercise influences mitochondrial function (47), suggesting that HIIT may be more effective in improving mitochondrial function and reducing apoptotic markers. Mitochondrial homeostasis requires HSP60, a mitochondrial chaperone protein (24). Increases in HSP60 following HIIT can prevent cardiomyocyte apoptosis by improving mitochondrial function and homeostasis. The effects of miR-206 on its downstream molecular target (HSP60) as well as apoptotic markers suggests that exercise-induced reductions in miR-206 can reduce apoptosis *in vitro*. Our *in vitro* data supports our hypothesis that the exercise-induced decreases in markers of cardiomyocyte apoptosis, and that increases in HSP-60 (especially with HIIT) may be mediated, at least in part, by reductions in miR-206 expression and blood glucose levels.

Diabetes-induced cardiomyopathy includes cardiac functional and morphological abnormalities that are accompanied by myocardial fibrosis, ventricular systolic and diastolic dysfunction, reduced ejection fraction, and impaired cardiomyocyte contractility (2). Regular exercise training promotes cardioprotective adaptations associated with improved cardiac function and structure (8, 13, 14). We used echocardiography to demonstrate that both exercise protocols improved left ventricular function by reducing end-diastolic and end-systolic parameters in STZ-induced diabetic rats. In addition, exercise training also improved ejection fraction in diabetes. Exercise training restores diabetic cardiac abnormalities in an exercise intensity-dependent manner. The superior effect of high-intensity exercise over moderate-intensity exercise on cardiac adaptations is well-documented in both human (48) and animal studies (49).

Hyperglycemia and disrupted energy metabolism are responsible, at least in part, for diabetes-related cardiac abnormalities. Given that both exercise training programs were associated with improvements in cardiac functional and morphological characteristics, it is reasonable to suggest that improvements of blood glucose and underlying cellular modifications induced by both exercise training, particularly HIIT, could improve cardiac function. It has also been suggested that exercise at a high intensity stimulates sympathetic activity, with the greater sympathetic activity in HIIT promoting cardiac contractile capacity and function (50). Our results are supported by findings that HIIT was more effective than moderate exercise training in improving systolic and diastolic function and VO_2peak_ in T2DM (51) and that HIIT improves VO_2peak_ and left ventricular remodeling more than moderate continuous training in patients with prior myocardial infarction and left ventricle systolic dysfunction (52) and also that HIIT improved cardiac structure and function in T2DM patients by increasing left ventricular wall mass, end-diastolic volume, and stroke volume after 12 weeks of exercise (53). In addition, HIIT has a superior effect on vascular endothelial function in patients with diabetes (29). However, some studies reported no differences in VO_2peak_ in patients with T2DM after HIIT and MICT (49). Nevertheless, the majority of studies suggest that the intensity of exercise is an important determinant in improving cardiac function in diabetic patients, and that moderate-intensity exercise may be insufficient to improve myocardial function.

Our study has some limitations that should be acknowledged: (1) We did not assess markers of the apoptotic cascade, such as caspase-9, caspase-3, caspase-8, Bax, Bak and p53. Future research should also measure the influence of different exercise programs on the expression levels of heart failure markers such as ANF, BNP, b-MHC, and (2) We tested LVEDD and LVESD using echocardiography, with no independent confirmation using cardiac tissue sections from the different experimental groups.

In conclusion, our study demonstrates that physical exercise has the potential to reduce plasma glucose levels and miR-206 expression. Suppression of miR-206 and the resulting positive regulation of HSP60 expression leads to improved cardiac function of diabetic rats following physical exercise. Although it has been established previously that MICT provides cardioprotection in diabetes, HIIT appears to stimulate even greater adaptations. Our findings suggest that HIIT should be preferred over MICT if the goal is to treat adverse effects related to diabetes including cardiomyopathy. Moreover, HIIT is a time-efficient exercise protocol compared with MICT.

## Data Availability Statement

The original contributions presented in the study are included in the article/supplementary materials, further inquiries can be directed to the corresponding authors.

## Ethics Statement

All animal procedures were carried out humanely and in accordance with the guidelines of Tehran University of Medical Sciences' Animal Care Committee (EC-00312).

## Author Contributions

MD, RA, AS, SG-F, and HZ participated to the conception and design of the study. MD, SG-F, and RA were responsible for testing. MD, RA, AS, SG-F, JH, and ND were responsible for data collection and statistical analysis. MD, AS, IL, UG, and HZ were responsible for writing and finalization of the manuscript. All authors contributed to manuscript and approved the submitted version.

## Funding

The authors acknowledge the support of the Deutsche Forschungsgemeinschaft (DFG, German Research Foundation) – Project number 491466077 and Open Access Publishing Fund of the University of Potsdam, Germany. This work was financially supported by Alzahra University.

## Conflict of Interest

The authors declare that the research was conducted in the absence of any commercial or financial relationships that could be construed as a potential conflict of interest.

## Publisher's Note

All claims expressed in this article are solely those of the authors and do not necessarily represent those of their affiliated organizations, or those of the publisher, the editors and the reviewers. Any product that may be evaluated in this article, or claim that may be made by its manufacturer, is not guaranteed or endorsed by the publisher.

## Abbreviations

HSP60: heat shock protein 60
PARP: poly ADP-ribose polymerase.
